# Benthic biofilm structure and function under abrupt flow changes

**DOI:** 10.1371/journal.pone.0327216

**Published:** 2025-07-23

**Authors:** Julie Anne Hope, Julia Kleinteich, Sabine Ulrike Gerbersdorf

**Affiliations:** 1 Institute for Modelling Hydraulic and Environmental Systems, University of Stuttgart, Stuttgart, Germany; 2 Scottish Oceans Institute, School of Biology, University of St Andrews, St Andrews, Fife, United Kingdom; 3 Center for Applied Geoscience, Eberhard Karls, University of Tübingen, Tübingen, Germany; INRA/Sorbonne University, FRANCE

## Abstract

Sediment accumulation reduces the capacity of dammed systems worldwide, therefore understanding sediment stability and transport within a reservoir is fundamental for sustainable management. Fluctuating hydrodynamics can alter the physical disturbance exerted on the sediment bed and can lead to substantial resuspension of bottom sediments and benthic biofilms. Removal of the biofilm can drastically alter the biochemical environment in the bed, and its ability to stabilize underlying sediments as microphytobenthic and bacteria communities are removed. In this experiment, an 8-week long hydraulic flume experiment was conducted to examine the response, adaptation and functionality of biofilms exposed to abrupt increases in flow typical of flow managed systems. Water and resuspended sediment from an oligotrophic reservoir in Germany, were used to develop biofilms on inserted flume cartridges. Developed biofilms were haphazardly distributed across two flow treatments: high bed shear stress (0.7 Pa) or low bead shear stress (0.1 Pa) for 28 days. Biochemical changes and biostabilization potential (adhesiveness) were examined over this period. Microphytobenthic biomass and composition, bacterial community diversity and extracellular polymeric carbohydrates/proteins were all initially altered by the abrupt increase in flow as the biofilm was stripped away. Biochemical properties largely recovered by the end of the experimental period (28-days) with recovery time and the degree of re-establishment dependent on the initial biofilm condition. However, sediment beds exposed to higher flows remained less stable, suggesting this functional role of the biofilm may take longer to reestablish itself after periods of higher flow. Findings suggest flow management has the potential to alter biofilm development and highlight the importance of protein content and microphytobenthic biomass in the recovery of biostabilization, Microphytobenthic diversity and carbohydrate content had less influence in the recovery of biostabilization. The findings may be useful to reservoir managers to manipulate flow to allow stable benthic biofilms to improve water quality and/or reduce infilling.

## Introduction

Dams and reservoirs can significantly impact the river continuum, altering the frequency and magnitude of floods and flow [1,2] as well as the retention and movement of sediments [3,4]. Sediment infilling of a reservoir negatively affects and limits dam operation, functionality and lifetime as storage capacity is reduced over time [5–7]. Sediment flushing, or reservoir drawdowns are typically used to remove accumulating sediment deposits and can be crucial for large catchments such as the Mekong [8] that can become sediment starved downstream [9]. These processes can be detrimental for downstream fauna when high loads of sediment and organic matter can smother them [10,11]. Flow management can alter phytoplankton community dynamics and lead to the development of harmful algal blooms within reservoirs [12,13], but flow changes can also alter benthic microbial community composition and diversity [14] and the development of benthic biofilms [15].

Benthic microbial organisms, the microphytobenthos (MPB) and bacteria, can secrete extracellular polymeric substances (EPS) containing carbohydrates and proteins that form a protective biofilm over the surface of a variety of sediment beds [16]. These biofilms play prominent roles in various functions, including carbon fixation, fueling food webs, cycling nutrients and underpinning biodiversity (for a more detailed review please see [17]). Benthic biofilms also play a significant role in mediating sediment resuspension as its formation on the surface protects underlying sediments, which in turn influences the bentho-pelagic exchange of particulates [17,18]. Biofilms also promote self-purification [19] and can control biogeochemical fluxes of pathogens, nutrients and pollutants into the water column preventing harmful algae blooms from occurring [20,21]. The influence of biological stabilization (biostabilization) on sediment dynamics has been recognized in many freshwater and marine systems [18,22,23], however, less is known about benthic biostabilization in reservoir systems. Understanding the influence of benthic biofilms on reservoir sediment behaviour is particularly relevant in times of global change and increasing water demand [13,24]. Evidence of biological cohesion of sediments can also help parameterize sediment transport models both within the reservoir in terms of when critical erosion thresholds, and the influence benthic biofilms on particulate settlement in downstream environments [25].

Previous studies have typically investigated biofilm structure and function under constant flow conditions, and the effects of abrupt hydrological changes (such as those in dammed systems) on benthic biofilms are poorly characterized. Biofilms that develop under higher flow velocities often exhibit greater bacterial diversity [14] and higher biostabilization [26], compared to those that develop under low flow conditions. Fast flow biofilms are often thinner, denser and more compact making them well suited to withstand greater physical disturbance [27], however, biofilm development and stabilization potential of the bed may be slower [28]. In contrast, biofilms developing in low flow often appear less compact and fluffier, with this loose material often easier to erode and slough-off under increasing physical disturbance [29]. These differences are due to the adaptation of the microbial community to the prevailing flow conditions with the physical disturbance from water flow influencing biofilm architecture, the community of micro-organisms and the degree of biostabilization [22,28,30] with typically greater stability under more physical disturbance observed [31]. Further evidence is therefore required to understand the resilience and adaptation of benthic biofilms when flow is suddenly increased as typically occurs in reservoir systems.

To address this gap in knowledge, this study aimed to assess the effects of abrupt flow increases on benthic biofilms that were initially developed under minimal flow (typical of reservoir systems). We hypothesized that the initial biofilm condition (the relative strength of the biofilm prior to flow alteration) would influence its response, adaptation and functionality under increased physical disturbance. It was expected that the MPB and the biofilm matrix (of EPS) would be physically removed as flow was initially increased. As the quantity and quality of MPB and bacteria on the surface and associated extracellular polymeric substances (EPS–carbohydrates and proteins) are key properties that influence biostabilization processes [17], their removal was expected to be associated with changes in biostabilization. Finally, it was anticipated that the adaptation and re-estabilishment of the benthic biofilms over time would coincide with the recovery of biostabilization.

## Methods

### Experimental design

Biofilms were developed under low flow conditions (flow velocity – 0.034 m s^-1^) in recirculating hydraulic flumes for an initial growth period of 28 days. Biofilms were assessed and grouped into strong and weak biofilms based on their adhesive capacity (see below), before being haphazardly exposed to either i) the same low flow conditions (control, flow velocity – 0.034 m s^-1^) or ii) high flow conditions (flow velocity = 0.15 m s^-1^). These treatments equated to typical low-flow conditions (~0.00–0.04 m s^-1^) measured during dry summer periods at the site, and a bed shear stress (BSS) of ~0.1 Pa, and a higher BSS of ~0.7 Pa, based on previous studies using the same system [28], velocities experienced near partially open sluice gates [32] ensuring that flows remained below the critical erosion threshold of the underlying particles. This simulated an abrupt flow increase (high flow) and a control flow (low flow) to allow sediment properties and the adhesive nature of the biofilm (as a proxy of biostabilization) to be measured over time in order to understand the loss, development and adaptation of the biofilms and their role in biostabilization.

### Experimental set up

Sediments were prepared by filling a total of 96 small cartridges (0.08m x 0.06m x 0.02m, Length x Width x Height, S1 File), with inert, abiotic glass beads similar in size to very fine to fine sand (100–200µm; Wentworth scale). This provided a fine, but cohesion-free substratum (e.g., free from microbial organisms and clay present in natural sediments that effect biological and physical cohesion respectively) to inoculate with a natural biofilm. Cartridges were positioned in the test section of six identical recirculating laboratory flumes. Sediment surfaces were aligned with the surface of a false floor in the flume (see S1 File for details [33]). Each flume (3m x 0.15m x 0.15m) allowed fully turbulent flow and constant shear stress over the test section area (1m), where 16 sediment cartridges were housed.

Unfiltered, resuspended water from the oligotrophic Schwarzenbach reservoir (SBR), Germany, was collected on 13^th^ July 2016 to inoculate the flumes with microorganisms. No permits were required by the reservoir provided authorization for access. Water was distributed equally (~200L) between the flumes and recirculated using BADU Eco touch pumps (Speck Pumpen, Neunkirchen am Sand, Germany). Temperature was maintained at ~15^o^C throughout the experiment by means of a cooling water circuit and flow velocity was monitored using mini-flow meters (Bürkert 8030, Ingelfingen, Germany) installed and calibrated to derive bed shear stress (<0.01 Pa minimum velocity) from the discharge rate [33]. Two parallel fluorescent tubes (Osram Biolux; 480–665nm) were calibrated using a high-resolution spectroradiometer [34] and secured 28.5 cm above the surface of the cartridges to produce a light intensity of 50 µE m^2^ s^-1^ at the sediment surface.

After the initial biofilm development under low flow, the adhesive capacity of the cartridges was examined using Magnetic Particle Induction (MagPI, please see method below). From this initial evaluation of sediment adhesion, biofilms were grouped into ‘weak/thin’ (adhesive capacity < 500mA) or ‘strong/thick’ biofilms (adhesive capacity > 500mA), with their adhesiveness coinciding with visible structural differences (thin, light and compact or thick dark and fluffy). These are hereafter referred to as strong and weak biofilms respectively. Water depth was reduced across all flumes to allow the careful placement of biofilm cartridges, with the biofilms (weak/strong) allocated haphazardly across the replicate flumes and allowed to acclimate for a further 24h after the water depth was reset.

### Sample collection

After cartridges were acclimated to the new flumes for 24h, sampling commenced (T0) from each biofilm condition (strong/weak, 3 replicates each) and the flow velocity in the high flow flumes were increased. Post flow-treatment samples were taken 24h after the flow change (T1) and periodically over the next 28 days (T3, T7, T14, T28) with each time point (T0-28) equating to the number of days after the change in flow. At each sampling point, the flow was carefully reduced to allow the sediment cartridges to be removed without disturbance. The variation in biostabilization and biofilm properties were evaluated from three randomly selected weak and strong biofilms in each individual (replicate) flume. A closed cartridge (no sediment) replaced each removed sediment cartridge to maintain a flat-bed in each flume. To account for the small-scale heterogeneity and natural patchiness of biofilms [35], five cores (0.3 cm^3^) were randomly collected, pooled and homogenized from within each cartridge for the extraction and determination of biochemical properties. This allowed an average to be calculated for each cartridge, with replicate cartridges taken from each of the flumes (three true replicates). Samples for DNA and chlorophyll a analysis were frozen immediately and stored at -20^**o**^C until processed with samples for algal community analysis fixed in 2% Lugol solution and stored in a cool dry space until cleaned. EPS proteins and carbohydrates were immediately extracted, with supernatants frozen at -20^**o**^C until processed following Gerbersdorf et al. [36].

### Characterizing the EPS matrix and the microbial community

Chlorophyll a/pheophytins were quantified (n = 72, from 6 replicate flumes, both biofilm conditions (strong/weak) and from each of the 6 days) following DIN 38 412/16 protocol, using 96% ethanol before and after acidification to quantify the microphytobenthic biomass corrected for degradation products [37]. EPS protein and carbohydrate fractions were determined spectrophotometrically using the modified Lowry method [38] and Dubois assay [39], respectively, from triplicate extracts.

Microphytobenthic cells for microscopic analysis and DNA were only processed from T0, T1, T7 and T28, due to cost and time constraints. Cells for microscopic identification were cleaned of organic material by boiling the samples in a 30% H_2_O_2_ solution followed by a 15% HCl solution to remove any carbonates. All samples were rinsed a minimum of four times in MilliQ water and the frustules embedded in Naphrax on slides (Northern Biological Supplies, England). Identification was conducted under a Zeiss Universal light microscope, phase and a Ph3-NEOFLUAR oil immersion objective x100 coupled to a 1.0 and 2.0 optivar and primarily followed Battarbee & Charles [40], Krammer & Lange-Bertalot [41–44], and Hofmann, Werum & Lange-Bertalot [45]. A minimum of 300 frustules were identified from each sample to species, where possible, and the relative abundance of each taxon recorded.

DNA was isolated from approximately 0.25–0.30 g of defrosted sediment, using a Nucleospin Soil extraction kit according to the manufacturer’s guidelines (Macherey and Nagel, Düren, Germany). DNA was extracted in duplicate and titrated to a standard working concentration of 50 ng µL^-1^. Automated Ribosomal Intergenic Spacer Analysis (ARISA) was performed on PCR amplification of the bacterial internal transcribed spaced (ITS) gene region. PCR amplification followed Kleinteich et al. [46], using the Q5® High fidelity 2x Master Mix polymerase (New England Biolabs, Ipswich, MA, USA) and the bacteria specific primer set S-D-Bact-1522-b-S-20 labelled at the 5’ end with the FAM fluorochrome and L-D-Bact-132-a-A-18 (Eurofins Genomics, Ebersberg, Germany), as described by Ranjard et al. [47]. Full details are provided in the supplementary material. Separation of fragments and analysis of peak intensity for the attained ARISA fragment lengths (AFLs) was conducted by Eurofins Genomics via Capillary electrophoresis on an ABI 3130 XL sequencing machine (Applied Biosystems, Foster City, CA, USA) using the LIZ1200 size standard and the ABI-G filter system. Data was processed as described in Wood et al. [48] prior to further statistical analysis.

### Measurements of biofilm/sediment-complex adhesion

A semi-automated Magnetic Particle-Induction (MagPI) system was used to reduce subjectivity due to observer differences [33]. MagPi utilizes ferrous particles that are added to three small intact surface areas of the sediment (6 mm^2^) of the cartridges (3 x technical replicates were averaged for each replicate cartridge to account for biofilm patchiness). An electro-magnet was positioned 4 mm above the ferrous particles and the magnetic force (mA) auto-increased incrementally over time. A microscopic camera captured images for later evaluation. The percentage of the ferrous particles removed as the magnetic force was incrementally increased was determined from binary images using ImageJ (see supplementary information for full method description) as a proxy of sediment adhesiveness. The magnetic force (at a given increment) required to significantly reduce the surface area covered by ferrous particles can then be used to calculate different metrics with the 50% and 75% removal of ferrous particles selected for further analysis.

### Statistical analysis

Euclidean distance matrices were constructed for the biochemical properties of the biofilms, while Bray-Curtis similarity matrices were used for the diatom and bacterial community data. The abundance of ARISA fragment lengths (AFLs) was used as an indicator of the number of bacterial species and are described as individual operational taxonomic units (OTUs) herein. As the bacterial OTUs do not indicate the number of a particular species within a sample, diversity was not calculated but richness was. Presence/absence of bacterial OTUs was extracted rather than species numbers [PRIMER, V6, 49]. To assess the influence of different biochemical properties on biostabilization (adhesiveness), the biochemical data (MPB biomass, bacterial numbers, MPB protein and carbohydrate contents), was treated using a dispersion weighting across the days before a Euclidean matrix was constructed on fourth-root transformed data. The matrices of community and biofilm property data were compared across the days, for initial biofilm condition (weak vs strong) and flow treatment (high vs low) in separate 3-way PERMANOVA tests using 9999 permutations [50] to determine the effects on diatom community composition, bacterial OTU richness and community composition, as well as biochemical properties of the system. PERMANOVA tests yield a Pseudo-F statistic and an associated p-value, with the threshold for statistical significance set at an alpha level of 0.05. All permanova output tables are provided in the supplementary material. Non-significant interaction terms were removed from the models, after comparing r^2^ values, leaving significant 2-way interactions. Principal components analysis plots (PCoA) were used to visualize the multivariate diatom community dataset, with SIMPER used to identify which diatom species contributed the most to the differences among groups. A canonical analysis of principal components (CAP) ordination constrained by the biochemical resemblance matrix was used to examine the collective effects of biochemical properties (Chl a, EPS-carbohydrate, EPS-protein, and OTU richness) on sediment adhesion (mA values). Diatom community data was not included in this CAP analysis as diatoms were only identified for a subset of the samples.. CAP analysis was conducted in Primer [PRIMER, V6, 49], however the final ordinations were produced in R4.2.3 using the vegan package [51].

## Results

### MPB biomass, EPS carbohydrates and proteins

**Fig 1:**
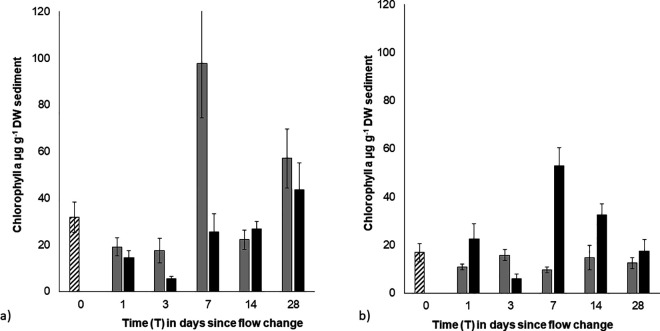
Mean chlorophyll a content of the surface sediment of a) initially ‘strong’ biofilms over time (0.3 cm^3^, ± SE, n = 3). **b)** initially ‘weak’ biofilms over time (0.3 cm^3^, ± SE, n = 3). Striped bars – initial pre-treatment values (0.1 Pa); grey bars – low BSS treatment (0.1 Pa); black bars – high BSS treatment (0.7 Pa).

Permanova tests detected a 3-way interaction between biofilm condition, flow and time on chl a content in the bed (Pseudo-F = 141.94, p < 0.001) indicating the combined effect of biofilm strength and flow changes over time (Fig 1A and 1B). For strong biofilms (Fig 1A), a significant reduction in MPB biomass (chl a content) was detected in under high flow (Strong - T1, T3) compared to pre-treatment (T0) MPB biomass levels (p < 0.001; Fig 1A) but by the end of the experiment (T28), MPB biomass had returned to pre-treatment levels (T0 = T28). In comparison, the initial increase in flow did not significantly alter the MPB biomass (chl a content) of weak biofilms from pre-treatment values (weak - T0 = T1, or from MPB biomass under low flow. By the end of the experiment (T28) the flow increase had a positive effect on MPB growth in weak biofilms, with greater biomass in biofilms subjected to high flow compared to those maintained under low flow (T7-T28; Fig 1B).

**Fig 2:**
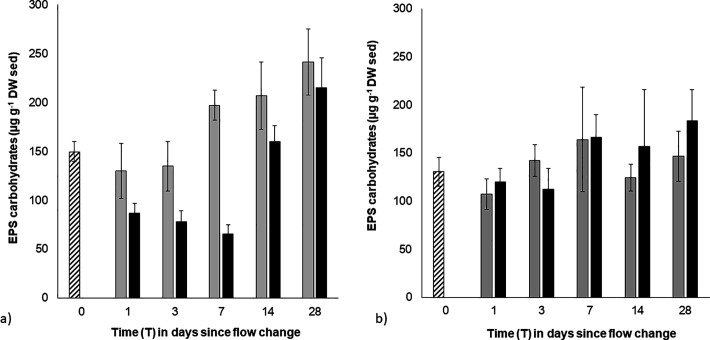
Mean EPS carbohydrate content of the surface sediment of a) initially ‘strong’ biofilms over time (0.3 cm^3^, ± SE, n = 3). **b)** initially ‘weak’ biofilms over time (0.3 cm^3^, ± SE, n = 3). Striped bars – initial pre-treatment values (0.1 Pa); grey bars – low BSS treatment (0.1 Pa); black bars – high BSS treatment (0.7 Pa).

The EPS carbohydrate content in initially strong and weak biofilms responded differently to increasing flow with a significant, 3-way interacting effect of biofilm strength, flow and time detected (Pseudo-F = 20.79, p < 0.001). This was largely attributed to the EPS carbohydrate content of strong biofilms being significantly reduced immediately after the flow increase (T0 > T1; t = 2.82, p < 0.001; Fig 2A). However, the final EPS carbohydrate content of strong biofilms was higher than pre-treatment values by the final day of the experiment (T28 > T0; p < 0.05) regardless of flow conditions.

In comparison to strong biofilms, the EPS carbohydrate content of initially weak biofilms was not significantly altered by the flow increase, however variable EPS carbohydrates contents, coinciding with biofilm patchiness, likely prevented the detection of any significant differences (Fig 2B). Overall, the EPS carbohydrate content played no significant role in the stability of weak biofilms, with EPS carbohydrates also correlating poorly with sediment stability measurements (r^2^ < 0.1).

**Fig 3:**
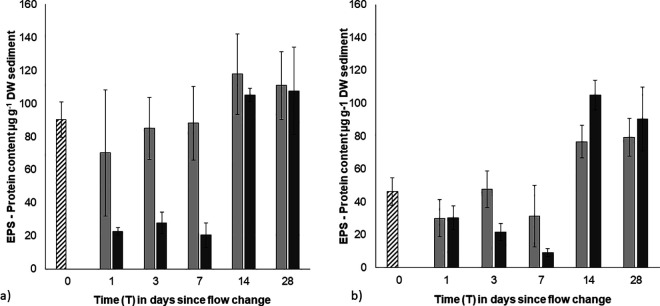
Mean EPS protein content of the surface sediment of a) initially ‘strong’ biofilms over time (0.3 cm^3^, ± SE, n = 3). **b)** initially ‘strong’ biofilms, over time (0.3 cm^3^, ± SE, n = 3). Striped bars – initial pre-treatment values (0.1 Pa); grey bars – low BSS treatment (0.1 Pa); black bars – high BSS treatment (0.7 Pa).

For the EPS protein content, there was also a significant, 3-way interacting effect of biofilm strength, flow treatment and day (Pseudo-F = 7.09, p < 0.001). However, in contrast to the EPS-carbohydrates, the initial protein content was significantly higher in stronger biofilms compared to weak biofilms (p < 0.01; T0, Fig 3A and 3B). EPS protein contents were variable in weak biofilms under both high and low flow, but flow-induced differences in EPS protein content were observed in strong biofilms in the days following the flow change (strong - T0 > T1-T7). There was less of an effect in weak biofilms. By the end of the experiment the final EPS protein contents of strong biofilms had recovered to starting values, irrespective of the flow treatment (strong – T0 = T14-T28; Fig 3A). In contrast, the protein content of the weak biofilms was significantly higher than the initial values by the end of the experiment regardless of flow (Fig 3B).

### Community dynamics

**Fig 4:**
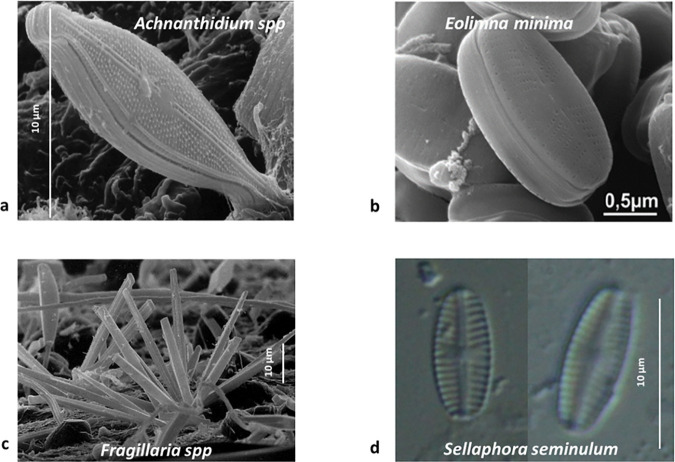
Low Temperature Scanning Electron microscope images of common diatoms observed during the experiment. **a)** *Achnanthidium spp*, valve length 5–20 μm. **b)** *Eolimna minima*, valve length 5–18 μm. **c)** *Fragillaria spp*, valve length 20–40 μm. Colonial species that often form filaments of cells mechanically joined to one another. **d)** *Sellaphora seminulum,* valve length <20 μm.

The *in situ* epilithon at the oligotrophic Schwarzenbach Reservoir (SBR) was sampled, with a total of 25 diatom species identified. MPB (diatom) community richness was similarly low (24 species) in the mesocosm experiment seeded with water from the SBR, but in some instances, the dominant taxa varied (the dominant species observed *in situ* are provided in the supplementary material). Four taxa were consistently detected across all samples, regardless of flow treatments, i.e., *Achnanthidium, Fragilaria, Eolimna* and *Sellaphora* genera (Fig 4)*. Achnanthidium minutissimum* (Kutz.) and *Achnanthidium minutissimum sp2* dominated the biofilms throughout (representing up to 98% of the community). While *Eolimna minima* accounted only for a very small portion of the epilithon in the SBR (0.2%) they were more abundant (0.4–8%) in the mesocosm experiment and particularly prevalent in strong biofilms.

While overall species richness and diversity was low, changes in the diatom community were still detected after the flow was altered and with time. Permanova detected two significant 2-way interactive effects on diatom community composition (the 3-way interaction was not significant and therefore not interpreted). The first significant, 2-way interaction (flow x initial condition) indicated the biofilm strength (strong/weak) may modulate the effects of flow change on the diatom community composition (Pseudo-F = 5.36, p < 0.01). The second significant 2-way interaction (flow x day) indicated that the diatom community changed differently over time under the different flow conditions (Pseudo-F = 4.03, p < 0.01), such that the differences in disturbance caused by flow, influenced the recolonization and community development over time. In strong biofilms, when diatoms were removed from the bed due to the flow increase, recolonization by other species was limited, and the same dominant species recolonized the bed such that the community composition was similar on T0 and T28. In contrast, under low flow the diatom community shifted over time becoming more dissimilar over time between T0 and T28.

**Fig 5:**
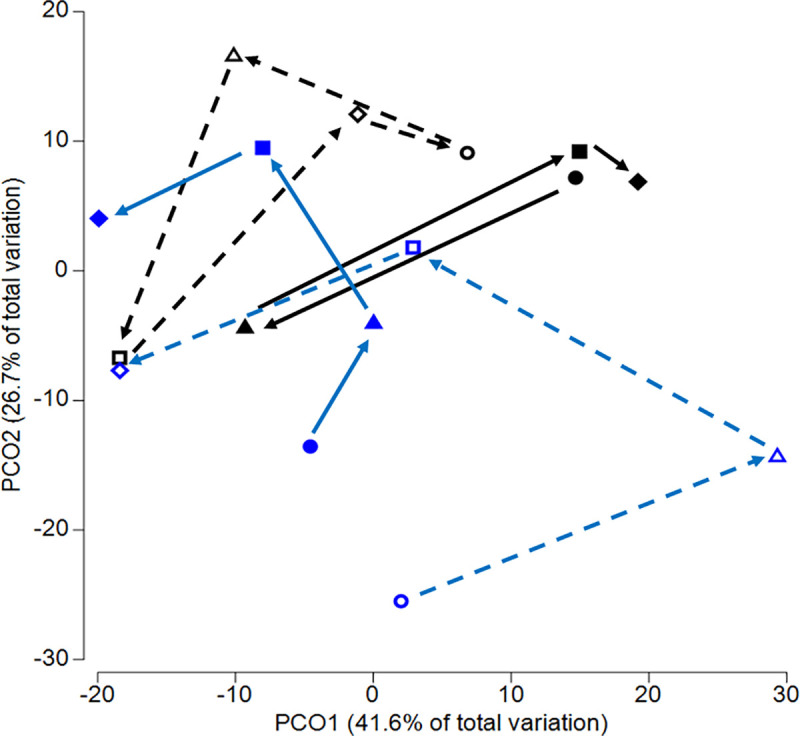
PCO ordination of the MPB diatom community based on the initial strength of the biofilm, flow treatment and days since the flow change. Closed symbols – strong biofilms; open symbols – weak biofilms. Black symbols – high BSS (0.7 Pa); blue symbols – low BSS (0.1 Pa). Symbol shape indicates the number of days since flow change: Circles – T0 (24 h pre-treatment); triangles – T1 (24 h post-treatment); squares – T7 (7 days post-treatment); diamonds – T28 (28 days post-treatment).

Visualization of the diatom community data supported the diatom community shifts, and the drivers identified above using permanova tests. The total variation explained by PCO axis-1 (41.6%) represented the change in community over time (day), with PCO axis-2 representing differences in the flow regime (high/low flow; Fig 5). In strong and weak biofilms (closed and open black circles), a change to high flow first increased dissimilarity (T0 to T1) with a shift from positive to negative values on axis 1. However, community dissimilarity then decreasing over time (T7 and T28) as the diatoms adapted to the higher flow conditions (Fig 5).

For diatom communities maintained under low flow (blue symbols), diatom communities were more dispersed across the ordination plot and became more dissimilar over time (Fig 5). While there was no flow change applied, there was an initial shift in the diatom community due to the disturbance caused by altering water depth to move cartridges. Under low flow, the community shifted in the opposite direction along axis 1 (from negative to positive) between T0 and T1, before shifting back along axis 1 by the end of the experimental period.

Similarity percentage analysis (SIMPER) based on Bray-Curtis similarity matrices identified the relative contribution of different taxa to these changes, and highlighted that differences were largely due to the removal of *Achnanthidium minutissimum* sp 2 under higher flow. The removal of this species contributed to the high dissimilarity across the days under high flow regardless of the biofilm condition (~70% dissimilarity for both strong and weak biofilms, Table 1). However, under low flow conditions, the abundance of *Achnanthidium minutissimum* sp 2 increased from T0 to T1 in strong biofilms. Dissimilarity then decreased over time, with diatom communities at the end of the experiment (T28), relatively similar to initial communities (T0), particularly in the strong biofilms (Table 1). In contrast the weak biofilm communities were less similar to the starting communities (T0) by the end of the experiment (T28), confirming the increase in other species present.

**Table 1:** SIMPER analysis identifying the percentage dissimilarity in the diatom community. The initial strength of the biofilm under different flow conditions between T0 (24 h pre-flow treatment) & T1 (24 h post treatment), and T0 & T28 (28 days post treatment). Comparisons with T7 were not included in the analysis to focus on the initial and final changes in community composition.

Initial biofilm strength	Flow condition	Dissimilarity T0 – T1	Dissimilarity T0 – T28
Strong	High	68%	10%
Strong	Low	39%	21%
Weak	High	72%	37%
Weak	Low	31%	30%

**Fig 6:**
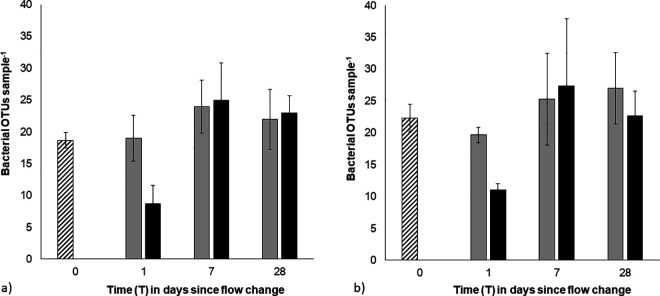
Mean number of bacterial OTUs detected in surface sediments of a) initially ‘strong’ biofilms over time (0.3 cm^3^, ± SE, n = 3). **b)** initially ‘weak’ biofilms over time (0.3 cm^3^, ± SE, n = 3). Striped bars – initial pre-treatment values (0.1 Pa); grey bars – low BSS treatment (0.1 Pa); black bars – high BSS treatment (0.7 Pa).

Bacterial community analysis (ARISA) identified between 4 and 48 different OTUs from the biofilm samples. While this may seem low for bacterial colonization, these communities were established on abiotic glass beads over a relatively short time, seeded from an oligotrophic system. Prior to the flow change (T0), there was no difference in the mean number of OTUs across strong and weak biofilms (Fig 6A and 6B). However, the mean number of bacterial OTUs decreased immediately after in the flow increase (T0 > T1, high flow) in both strong and weak biofilms, suggesting bacteria were removed during the erosion of the surface biofilm. This effect was temporary, with bacterial OTU numbers similar to starting values by day 7 which was maintained until the end of the experiment (T0 = T7-T28; Fig 6A and 6B). Permanova tests revealed a 2-way interaction between the biofilm strength and time (Pseudo-F 2.91, p < 0.05).

No 3-way interactive effects of flow, biofilm condition and time on bacterial community composition were detected due to the high variability and low statistical power for a 3-way analysis. However, bacterial community composition varied with the initial biofilm condition over time (biofilm condition x time; Pseudo-F = 1.46, p = 0.01), indicating the composition change of OTUs with time differed across the biofilm conditions. There was an additional main effect of flow (Pseudo-F = 2.33, p < 0.05) indicating flow influences community composition in a consistent way, namely differences in OTU numbers at T1 immediately after the flow increase, but flow does not modulate time-dependent differences among biofilm conditions.

### Biostabilization potential

**Fig 7:**
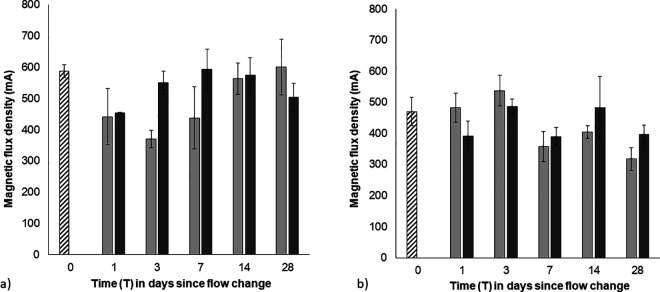
Mean magnetic force required to remove 75% of the ferrous particles from the sediment surface of a) initially ‘strong’ biofilms over time (±SE, n = 3). **b)** initially ‘weak’ biofilm over time (±SE, n = 3). Striped bars – initial pre-treatment values (0.1 Pa); grey bars – low BSS treatment (0.1 Pa); black bars – high BSS treatment (0.7 Pa). Mean Magnetic force required to remove ferrous particles from abiotic glass beads (prior to the development of a biofilm) was 100 mA.

The greater loss of MPB biomass (chl a) and EPS properties from strong biofilms immediately after the flow increase coincided with a significant reduction in sediment adhesion at high flow (Fig 7A). While a correlation between MPB biomass and sediment adhesion would be expected the relationship was not significant when considering strong (r^2^ < 0.1) and weak (r^2^ < 0.1) biofilms separately (as there are too few data points). However, as the strong and weak biofilms represent a gradient of biofilm growth, when grouped together this resulted in a significant correlation between MPB biomass (chl a) and sediment adhesion (r^2^ = 0.33, p < 0.05). In addition, EPS-carbohydrates did not correlate with adhesion in strong and weak biofilms (r^2^ < 0.1), but EPS-proteins, was significantly correlated with adhesion, particularly in strong biofilms (r^2^ = 0.52, p < 0.001).

In strong biofilms, the magnetic force required to remove particles was reduced from 588 ± 20 mA (strong - T0), to 454 ± 3 mA immediately after the flow increase (T1) (Fig 7A). Sediment adhesion then increased under high flow, with higher values for day 3 and 7. In contrast, the adhesiveness of weak biofilms was more variable over time but had decreased towards the end of the experiment regardless of the flow conditions; from 470 ± 46 mA (T0) to 397 ± 29 mA (T28) under high flow, and from 469 ± 46 mA (T0) to 318 ± 37 mA (T28 in low flow (Fig 9B). Despite the variability over time, by the end of the experiment weak biofilms were still more adhesive under high flow than under low flow conditions. Permanova tests detected a 3-way interaction between biofilm condition, flow and time (3-way interaction; Pseudo-F = 17.7, p < 0.001). This highlights that the initial biofilm condition with sediment adhesion increasing over time in response to the sustained increase in flow. This was predominantly related to the heightened adhesiveness of strong biofilms under high flow in the week after the flow increase (T3 and T7), with no difference in sediment adhesion under high and low flow in strong biofilms by the end of the experiment (T28, Fig 7A). In contrast, sediment adhesion across high and low flow treatments were variable over time across weak biofilms (Fig 7B) weak biofilms were significantly more adhesive under high flow by the end of the experiment.

**Fig 8:**
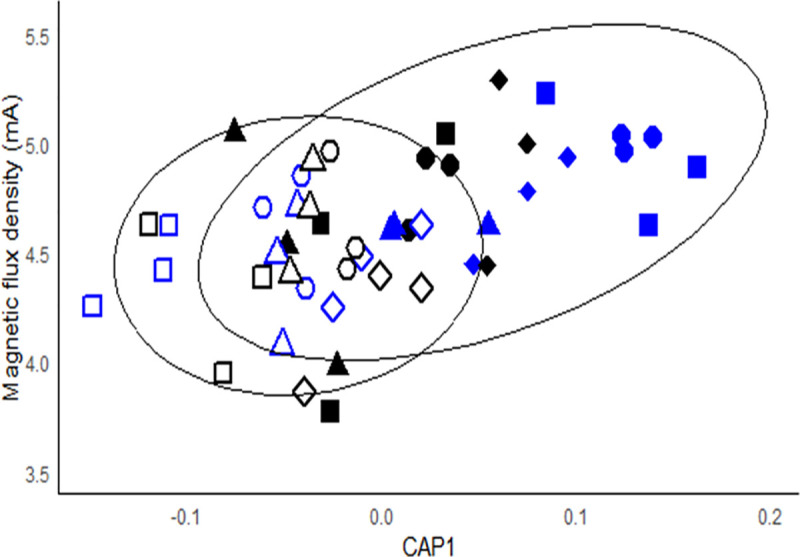
CAP ordination constrained by the biochemical resemblance matrix reveals the extent to which biofilm biochemical composition (EPS, Chl a, proteins) influences sediment adhesion taken as a proxy of biostabilization (mA values). The Euclidean matrix contained the number of bacterial OTUs, MPB biomass (chl a), EPS proteins and carbohydrate data (m = 4, r^2^ = 0.24, p < 0.01). A dispersion weighting was applied using the factor ‘day’ and all data was fourth root transformed prior to analysis. closed symbols – strong biofilms; open symbols – weak biofilms. black symbols – high BSS (0.7 Pa); blue symbols – low BSS (0.1 Pa). Symbol shapes indicate the day number: circles – T0 (24h pre-treatment); triangles – T1 (24h post-treatment); squares – T7 (7 days post-treatment); diamonds – T28 (28 days post-treatment). Only days 0, 1, 7 and 28 were used as this allowed the bacteria OTU data to be included in the analysis as OTUs were not quantified for day 3 or 14. Ellipses represent the 95% confidence intervals for strong and weak biofilms.

The 2D CAP plot (Fig 8) that explores the collective effects of biochemical components (Chl a, EPS-carbohydrates, EPS-proteins, OTU richness) illustrated the while sediment adhesion was significantly correlated to the matrix of measured biochemical properties (MPB biomass, EPS protein and EPS carbohydrates), only 24% of the variance was explained (p < 0.01), driven mainly by the effect of EPS-protein. This suggests other factors may play a significant role in sediment adhesion after a flow change. The strong and weak biofilms were clustered along the diagonal (ellipticals; Fig 8). Strong biofilms, subjected to high flow (black solid circles), became more dispersed after the flow change coinciding with the initial reduction of adhesiveness (T1) followed by higher adhesiveness over time compared to those kept at low flow. The stability/adhesiveness of weak biofilms was less variable regardless of flow conditions with points clustered closer together.

## Discussion

This study demonstrates two important aspects regarding biostabilization in reservoir systems. Firstly, biofilms growing on and within fine sediments under a low/moderate hydraulic regime are impacted by abrupt increases in discharges, and this has the potential to influence the functional role of biofilms in sediment adhesion. The biofilms had little resistance to moderate increases in physical disturbance caused by increasing flow, with biofilm components stripped off and the bed stability significantly reduced. Secondly, the adaptation and response of the biofilm-sediment complex was somewhat dependent on the initial biofilm structure and composition as well as time after the flow change was initiated. This has implications for biofilm formation in reservoirs between periods when flow is increased such as when sluice doors are opened.

Our findings support the general consensus that hydrodynamic conditions experienced during biofilm growth periods dictate their stability and adhesion properties [52], but additionally that when that flow is increased the biofilm will adapt. This has previously been observed in flow cell research, where higher flow can bring more nutrients and oxygen to the surface of the biofilm stimulating growth [53]. However, these episodic and abrupt increases in flow may significantly reduce benthic MPB and bacterial biomass, change the community of microorganisms and reduce EPS carbohydrate and protein content, which together biostabilize the bed. Overall, the structural properties of the biofilms (EPS carbohydrate/protein content and MPB biomass levels) returned to their previous state within 28 days, but their biostabilization functionality did not recover as quickly. This suggests sediments may be less adhesive reducing their ability to restabilize the bed following flow changes. This has implications for the mobility of reservoir sediments depending on the frequency and intensity of hydraulic changes imposed. In managed systems, biofilm development periods may extend well beyond our 28-day experimental period and will be dictated by the management needs, highlighting the need for further investigations over longer timescales with repeated flow variations.

There are complex interactions and feedback processes between biofilm properties, local hydrodynamic conditions and biostabilization [22,52], leading to biofilms that vary in distribution and structure at different temporal and spatial scales [54,55]. When biofilms develop under low flow/stagnant conditions, and are then subjected to a sudden increase in flow velocity, they may become patchier, with remaining patches serving as sources of recolonization. Fluffy, protruding sections of the biofilm along with the basal layers can be eroded altering EPS components of the biofilm matrix and stabilization potential [56]. This compromises biofilm integrity [57], exposing underlying bare sediment. This phenomenon was clearly visible in the thicker, strong biofilms (S1B Fig in S1 File top cartridge) suggesting that once a critical erosion threshold has been passed, strong biofilms are stripped of their basal biofilm layers. In contrast, erosion of weak, thinner biofilms did not expose bare sediment, implying their compact basal layers remained (Fig 1B bottom cartridge). The formation of thinner, denser films under higher flow conditions has been observed in both fluvial systems [56] and biofilm flow cell studies [58] suggesting they are structurally better adapted to increased physical disturbance. This may be due to their compact structure, which reduces surface roughness and friction with the overlying water compared to the fluffier, strong biofilms, allowing them to persist under higher flow conditions.

Nutrient availability influences biofilm architecture up to a certain thickness [29], but beyond a critical threshold, the biofilm’s mechanical resistance to shear stress becomes more important [56]. Biofilms that develop under low physical stress (e.g., low flow) are less adapted to withstand sudden increases in shear stress. However, once high flow erodes the biofilm back to its basal layers, the enhanced water flow can increase nutrient transfer into the bed, promoting biofilm regrowth. This may explain the increase in diatom species richness in the weak biofilms under high flow by the end of the experiment.

Maintaining adequate flow at key times of the year could help mitigate water quality issues, by promoting benthic biofilm stability, while prolonged periods of extremely low flow or stagnation should be avoided to prevent deep biofilm erosion. Conversely, in systems where sediment infilling is a concern, slower biofilm regrowth may be preferable as it results in less stable sediment that can then be flushed out as biostabilization would be unlikely to withstand large flushing events, where flows can often exceed 100 Pa [59]. In this case, biofilm might still stabilize the edges of the former river channel and restrict the overall flushing success.

The initial structural differences in the biofilm influenced the biofilm adaptation to the new flow regime, affecting both biochemical components and adhesiveness and consequently sediment stability over time. Understanding biofilm development and structure before reservoir flushing or drawdown is essential for effective system management [60]. For instance, if water quality is a concern, allowing benthic biofilms to develop under slightly higher flow conditions, may enhance their recovery bed stabilization, reducing sediment resuspension and improving water quality through natural filtration.

The absence of larger motile diatoms, which are known to exude copious amounts of EPS carbohydrates into their environment [16] may explain the lack of correlation between EPS carbohydrates and stability measurements suggests the metabolic overflow of carbon from the microphytobenthic organisms, was not the driving component of sediment adhesiveness. While carbohydrates can be the major component in EPS, EPS also contains various proteins, enzymes and DNA which reflect differences in the community of microorganisms present [61]. EPS proteins often correlate positively with bacterial cell numbers and biostabilization in freshwater systems [62] and protein production can be stimulated by the interactions between the MPB and bacteria [63,64].

Bacteria play a role in biofilm stabilization by degrading and producing extracellular proteins that facilitate adhesion to sediment particles [65]. The observed increase in EPS protein aligns with findings of Araújo and colleagues [57] who reported enhanced cell–surface and cell–cell attachment under higher flow conditions in biofilm flow cell reactors linked to protein synthesis. These results underscore the importance of considering both EPS carbohydrate and protein fractions in reservoir systems and supports previous freshwater studies [66]-which found EPS proteins to have a stronger influence on biostabilization.

Flow increases removed diatom cells from the bed, regardless of the initial biofilm stability, yet *Achnanthidium* spp remained dominant throughout the experiment. As a pioneer species, *Achnanthidium* thrives in early biofilm formation [67] and low nutrient environments [68] making its dominance unsurprising in the oligotrophic waters of the SBR. While known for their resilience to high disturbance [69] *Achnanthidium* can also dominate low flow systems [70] highlighting its adaptability to varying conditions This explains their dominance during low flow conditions, as well as their ability to re-colonise the bed at higher physical disturbance.

The slight increase in *E. minima and F. pararumpens* and their proportional increase in strong biofilms during the experiment, may be related to their ability to attach via stalks and to form stack-like colonies within a biofilm [43]. This enables them to colonize new substratum quickly under high physical disturbance [69,71]. These species are often observed as biofilms begin to develop and their short development cycles enable them to proliferate in fluvial systems [72]. These characteristics give diatoms an advantage when flow conditions are altered [71], and other species are displaced. Despite the abrupt changes to the biophysical properties of the bed and stability, the community of microorganisms within biofilms recovered relatively quickly suggesting that both diatoms and bacteria can adapt to changing hydrodynamic conditions, by developing the biofilm to stabilize themselves and allow biomass to increase [71,73].

The response of biofilms to flow changes will also be influenced by other local environmental conditions not addressed in this study such as increasing nutrient loads [71,73]. By using cohesionless glass beads, we removed the influence physical cohesion to focus on biostabilization, but we know that biostabilization can differ depending on sediment type [68], with cohesiveness often increasing in the presence of mud and clay [56,74]. Furthermore, biostabilization may be greater in spring due to light and temperature driven increases in benthic primary productivity and biofilm growth [26] so flushing may be more effective and ecologically beneficial at particular times of the year. Additionally, variable light and flow conditions, nutrient availability and/or prey-predator relations may also influence the role of benthic biofilms in sediment stabilization [65,67,75] warranting their consideration in future studies.

## Conclusion

This study has demonstrated the potential for different hydrological regimes to influence the development, adaptation and stability of sediments within managed reservoir systems, which will in turn affect the biostabilization of the bed. While several complex mechanisms of biostabilization remain poorly understood, microphytobenthic and bacterial biofilms are a critical consideration in reservoir management, as the mediation of resuspended biochemical and lithogenic material can affect dam operations, sediment accumulation and water quality. Assessing the growth forms and characteristics of reservoir microbial communities can allow manager to better understand the potential feedback between hydrological management on biostabilization and sediment transport. Given the escalating pace of dam construction in many regions for water availability and the decommissioning of dams globally, this knowledge is vital.

## Supporting information

S1 File Supplementary methods. Additional methodological details of the flume/cartridge set up, adhesion measurements (MagPi), ARISA and *in situ* microphytobenthic community.
(DOCX)

S1 Table Permanova results. Permanova output tables for measured variables across days, biofilm strength and flow conditions.
(DOCX)
