# Supplementary material for: Benthic biofilm structure and function under abrupt flow changes
Source: PLoS One. 2025 Jul 23;20(7):e0327216. doi: 10.1371/journal.pone.0327216 (PMC12286397; doi:10.1371/journal.pone.0327216)
Supplement: S1 File — Additional methodological details of the flume/cartridge set up, adhesion measurements (MagPi), ARISA and in situ microphytobenthic community. (DOCX) [file pone.0327216.s001.docx]

# S1 File. Supplementary Methods

*Flume set up and Magnetic Particle Induction (MagPI)*

The magnetic force (mA) of an electro-magnet positioned 4mm above the surface was increased in 16 pre-defined increments up to 1170 mA. The system is automated, and a microscopic camera captures images of the biofilm surface at each increment for later evaluation of ferrous particle removal from the biofilm surface. These images are converted to binary images of ferrous particles (black) on sediment surface (white background) using thresholding. The percentage cover throughout the incremental steps can then be evaluated to determine the magnetic force required to significantly reduce the surface area covered by ferrous particles (See Thom et al., 2015).


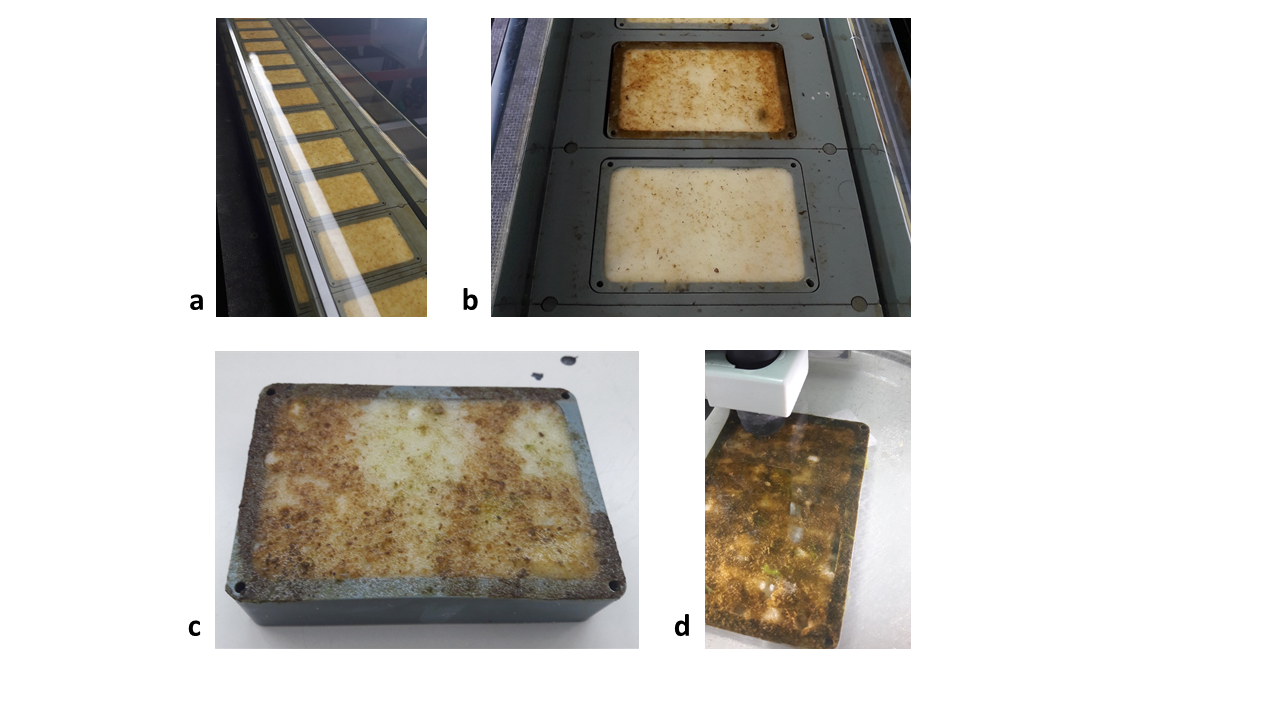


**Fig S1: a)** Biofilms were initially grown in individual cartridges across 6 replicate flumes. **b)** Biofilms were separated into ‘strong’ (top) and ‘weak’ (bottom) biofilms based on their initial stability at the end of the 28-day growth period. **c)** Patchy distribution of the surface biofilm of strong biofilms was visually apparent after the increase in flow. **d)** The adhesive capacity of biofilms tested in water at the end of the experiment using the MagPI system.

*Automated ribosomal intergenic spacer analysis (ARISA)*

Automated rRNA intergenic spacer analysis (ARISA) is a sensitive and high-throughput fingerprinting technique which is based on the heterogeneity in the length of the intergenic transcribed spacer (ITS) region between the 16S and 23S ribosomal genes (Wood et al., 2008). ARISA involves the use of a fluorescently labelled oligonucleotide primer, in this case a phosphoramidite dye fluorochrome, FAM (6-carboxyfluorescein) and PCR amplification of the total community DNA. Only automated fragment lengths (AFLs) greater than 200 bp length were regarded to be true intergenic transcribed spacer signals with signal intensities <200 fluorescence units (FU) discarded as background noise. Filtered data were input into a custom AFL binning script (Ramette, 2009) in R software and assigned to bins of 2 base pairs (± 1 base pairs (bp)) for fragments < 700 bp lengths, bins of 3 from 700 - 1000 bp length and bins of 5 bp for fragments > 1000 bp length to account for small shifts in the AFLs that can lead to the overestimation of species diversity. The final output was transformed into a peak area versus sample matrix, with each ‘peak’ representing an independent operational taxonomical unit (OTU) (Brown et al 2005; Hewson & Fuhrman, 2006). All OTUs were transformed to presence/absence data prior to statistical analysis. The total number of OTUs was calculated across the different samples. PERMANOVA tests of the total bacterial OTUs and community composition across the factors were performed based on Bray-curtis similarity resemblance matrices between samples.

*In situ microphytobenthic community – Schwarzenbach Reservoir*

*Achnanthidium minutissimum sp1* and *sp2* are both sessile diatoms, often found attached to substrates via stalks. These species dominated the original epilithon from field samples (43%, data not presented) and the mesocosms throughout our experiment regardless of treatments. Other species identified from the field samples included *Tabellaria flocculosa* 23% (rarely observed in the experiment) and *Fragilaria gracilis* 11% (present in experiment). *Fragilaria* is a larger (~50-several hundred µm) non-motile diatom of long-stretched thin needle-like morphology. This guild also includes morphologically similar species such as *Nitzschia*, which are typical of low flow conditions, as they are susceptible to high physical disturbance (Passy, 2007).
