# Supplementary material for: Benthic biofilm structure and function under abrupt flow changes
Source: PLoS One. 2025 Jul 23;20(7):e0327216. doi: 10.1371/journal.pone.0327216 (PMC12286397; doi:10.1371/journal.pone.0327216)
Supplement: S1 Table — Permanova output tables for measured variables across days, biofilm strength and flow conditions. (DOCX) [file pone.0327216.s002.docx]

**S1 Table: PERMANOVA results table for MPB biomass (chl a), EPS-carbohydrates, EPS-proteins, diatom community composition, bacterial OTU richness, bacterial OUT community composition and sediment adhesion.** df: degrees of freedom; SS: sum of squares; MS: mean sum of squares; Pseudo-F: F value by permutation, P(perm): p-values based on >999 permutations with P(MC); p-values based on Monte Carlo random sampling when possible permutations are low (<999); Perms: number of permutations run; St: strength of biofilm (strong/weak); fl: flow (high/low); da: days (T0-T28).

| **MPB biomass (Chl a)** | | |  |  |  |  |  |
| --- | --- | --- | --- | --- | --- | --- | --- |
| **Source** | **df** | **SS** | **MS** | **Pseudo-F** | **P(perm)** | **perms** | **P(MC)** |
| st | 1 | 1.2413 | 1.2413 | 5.180 | 0.07 | 9681 | 0.0001 |
| fl | 1 | 0.032381 | 0.032381 | 0.164 | 0.73 | 9710 | 0.07 |
| da | 5 | 3.3771 | 0.67541 | 329.740 | **0.0001** | 9939 | 0.71 |
| st x fl | 1 | 1.6347 | 1.6347 | 5.623 | 0.06 | 9835 | 0.0001 |
| st x da | 5 | 1.1981 | 0.23961 | 116.980 | **0.0001** | 9940 | 0.0001 |
| fl x da | 5 | 0.98437 | 0.19687 | 96.115 | **0.0001** | 9952 | 0.07 |
| **st x fl x da** | 5 | 1.4537 | 0.29073 | 141.940 | **0.0001** | 9952 | 0.0001 |
| Res | 48 | 0.098319 | 0.0020483 |  |  |  |  |
| Total | 71 | 10.02 |  |  |  |  |  |

**EPS-carbohydrates**

| **Source** | **df** | **SS** | **MS** | **Pseudo-F** | **P(perm)** | **perms** | **P(MC)** |
| --- | --- | --- | --- | --- | --- | --- | --- |
| st | 1 | 0.012377 | 0.012377 | 0.076 | 0.79 | 9693 | 0.80 |
| fl | 1 | 0.39203 | 0.39203 | 3.512 | 0.12 | 9683 | 0.12 |
| da | 5 | 2.023 | 0.4046 | 68.960 | **0.0001** | 9944 | 0.0001 |
| st x fl | 1 | 0.48098 | 0.48098 | 3.943 | 0.10 | 9843 | 0.10 |
| st x da | 5 | 0.8156 | 0.16312 | 27.802 | **0.0001** | 9947 | 0.0001 |
| fl x da | 5 | 0.55819 | 0.11164 | 19.028 | **0.0001** | 9943 | 0.0001 |
| **st x fl x da** | 5 | 0.60983 | 0.12197 | 20.788 | **0.0001** | 9959 | 0.0001 |
| Res | 48 | 0.28162 | 0.0058671 |  |  |  |  |
| Total | 71 | 5.1736 |  |  |  |  |  |

**EPS-Proteins**

| **Source** | **df** | **SS** | **MS** | **Pseudo-F** | **P(perm)** | **perms** | **P(MC)** |
| --- | --- | --- | --- | --- | --- | --- | --- |
| st | 1 | 1.7115 | 1.7115 | 22.904 | **0.01** | 9688 | 0.01 |
| fl | 1 | 1.4698 | 1.4698 | 3.841 | 0.11 | 9686 | 0.10 |
| da | 5 | 7.8885 | 1.5777 | 268.060 | **0.0001** | 9957 | 0.0001 |
| st x fl | 1 | 0.39559 | 0.39559 | 9.476 | 0.03 | 9837 | 0.03 |
| st x da | 5 | 0.37363 | 0.074726 | 12.697 | **0.0001** | 9947 | 0.0001 |
| fl x da | 5 | 1.9133 | 0.38267 | 65.018 | **0.0001** | 9959 | 0.0001 |
| **st x fl x da** | 5 | 0.20873 | 0.041745 | 7.093 | **0.0001** | 9955 | 0.0001 |
| Res | 48 | 0.28251 | 0.0058856 |  |  |  |  |
| Total | 71 | 14.244 |  |  |  |  |  |

**Diatom community composition**

| **Source** | **df** | **SS** | **MS** | **Pseudo-F** | **P(perm)** | **perms** | **P(MC)** |
| --- | --- | --- | --- | --- | --- | --- | --- |
| st | 1 | 390.69 | 390.69 | 1.22 | 0.35 | 839 | 0.36 |
| fl | 1 | 739.01 | 739.01 | 0.89 | 0.55 | 838 | 0.50 |
| da | 3 | 1101.7 | 367.22 | 1.79 | 0.15 | 9941 | 0.18 |
| **st x fl** | 1 | 1100.5 | 1100.5 | 5.36 | **0.01** | 9949 | 0.01 |
| st x da | 3 | 963.82 | 321.27 | 1.56 | 0.218 | 9949 | 0.24 |
| **fl x da** | 3 | 2484.2 | 828.06 | 4.03 | **0.01** | 9958 | 0.01 |
| Res | 3 | 616.37 | 205.46 |  |  |  |  |
| Total | 15 | 7396.3 |  |  |  |  |  |

**Bacterial OTU richness**

| **Source** | **df** | **SS** | **MS** | **Pseudo-F** | **P(perm)** | **perms** | **P(MC)** |
| --- | --- | --- | --- | --- | --- | --- | --- |
| st | 1 | 3487.3 | 3487.3 | 1.06 | 0.35 | 840 | 0.41 |
| **fl** | 1 | 4554.2 | 4554.2 | 2.01 | 0.06 | 838 | **0.03** |
| da | 3 | 16501 | 5500.5 | 2.49 | **0.0001** | 9839 | 0.0001 |
| st x fl | 1 | 1772.3 | 1772.3 | 0.92 | 0.54 | 9940 | 0.55 |
| **st x da** | 3 | 9880.8 | 3293.6 | 1.49 | **0.01** | 9845 | 0.02 |
| fl x da | 3 | 6791.5 | 2263.8 | 1.03 | 0.43 | 9837 | 0.42 |
| Res | 32 | 70608 | 2206.5 |  |  |  |  |
| Total | 47 | 119400 |  |  |  |  |  |

**Bacterial OTU community composition**

| **Source** | **df** | **SS** | **MS** | **Pseudo-F** | **P(perm)** | **Perms** | **P(MC)** |
| --- | --- | --- | --- | --- | --- | --- | --- |
| st | 1 | 3487.3 | 3487.3 | 1.08 | 0.36 | 840 | 0.39 |
| **fl** | 1 | 4554.2 | 4554.2 | 2.33 | 0.06 | 840 | **0.01** |
| da | 3 | 16355 | 5451.5 | 2.47 | **0.0001** | 9861 | 0.0001 |
| st x fl | 1 | 1772.3 | 1772.3 | 0.75 | 0.72 | 9908 | 0.72 |
| **st x da** | 3 | 9690.5 | 3230.2 | 1.46 | **0.01** | 9817 | 0.03 |
| fl x da | 3 | 5873.9 | 1958 | 0.89 | 0.73 | 9828 | 0.68 |
| Res | 32 | 70608 | 2206.5 |  |  |  |  |
| Total | 47 | 119400 |  |  |  |  |  |

**Sediment adhesion**

| **Source** | **df** | **SS** | **MS** | **Pseudo-F** | **P(perm)** | **perms** | **P(MC)** |
| --- | --- | --- | --- | --- | --- | --- | --- |
| st | 1 | 2111.9 | 2111.9 | 0.49544 | 0.54 | 9708 | 0.52 |
| fl | 1 | 8346.6 | 8346.6 | 3.8413 | 0.11 | 9650 | 0.11 |
| da | 5 | 59085 | 11817 | 71.268 | **0.0001** | 9936 | 0.0001 |
| st x fl | 1 | 11560 | 11560 | 3.9383 | 0.10 | 9854 | 0.10 |
| st x da | 5 | 21314 | 4262.8 | 25.709 | **0.0001** | 9941 | 0.0001 |
| fl x da | 5 | 10864 | 2172.8 | 13.104 | **0.0001** | 9942 | 0.0001 |
| **st x fl x da** | 5 | 14677 | 2935.3 | 17.703 | **0.0001** | 9959 | 0.0001 |
| Res | 48 | 7958.9 | 165.81 |  |  |  |  |
| Total | 71 | 135920 |  |  |  |  |  |
